# Hyperbaric Oxygen Therapy Ameliorates Olanzapine-Induced Hypolocomotion in a Rat Model

**DOI:** 10.3390/life14111482

**Published:** 2024-11-14

**Authors:** Ahmad Altarifi, Linah Arab, Rasha Al-Azaizeh, Batool Khataybeh, Muath Q. Al-Ghadi, Mohammad Khalifeh

**Affiliations:** 1 Department of Pharmacology, Faculty of Medicine, Jordan University of Science and Technology, P.O. Box 3030, Irbid 22110, Jordan; aaaltarifi@just.edu.jo; 2 Department of Veterinary Basic Sciences, Jordan University of Science and Technology, P.O. Box 3030, Irbid 22110, Jordan; leena.msa933@gmail.com (L.A.); rasha.alazaizeh@gmail.com (R.A.-A.); 3 Department of Nutrition and Food Technology, Jordan University of Science and Technology, P.O. Box 3030, Irbid 22110, Jordan; khataibehbatool@gmail.com; 4 Department of Zoology, College of Science, King Saud University, P.O. Box 2455, Riyadh 11451, Saudi Arabia; malghadi@ksu.edu.sa

**Keywords:** olanzapine, locomotor activity, hyperbaric oxygen therapy, total antioxidant capacity

## Abstract

Olanzapine (OLZ) is a commonly prescribed drug for the treatment of schizophrenia and related disorders. However, OLZ use is associated with several adverse effects, including decreased locomotor activity and increased body weight. While the majority of studies have directed their focus towards managing the metabolic side effects of OLZ, there has been limited attention given to the effects on locomotor activity. This study aimed to investigate the potential therapeutic effect of hyperbaric oxygen therapy (HBOT) in alleviating OLZ-induced locomotor impairment in female Sprague Dawley rats. Subjects were divided into four groups: control rats (CR), HBOT, OLZ, and HBOT + OLZ. In addition to behavioral effects, we also evaluated the total antioxidant capacity (TAC) of rats’ brain tissue to demonstrate the maintenance of OLZ effectiveness in improving antioxidant status during the intervention using a rotarod device to measure locomotor activity and coordination. Results showed that HBOT effectively counteracted the hypolocomotion produced after OLZ administration. Moreover, HBOT did not result in a decrease in TAC in brain tissue, which is linked to OLZ treatment effectiveness. Therefore, our results suggest that HBOT may represent a promising non-pharmacological approach to improving locomotor and motor coordination impairments associated with OLZ treatment.

## 1. Introduction

Schizophrenia is a debilitating and chronic mental illness, characterized by positive symptoms (such as hallucinations, delusions, and disorganized speech and behavior) as well as negative symptoms, such as apathy, anhedonia, and poor attention [1]. Cognitive symptoms such as memory and attention deficits are also commonly observed in patients with schizophrenia [2]. Antipsychotics are the mainstay in the treatment of schizophrenia and other mental disorders. They are divided into first-generation (or typical) and second-generation (atypical) classes. Among the atypical antipsychotics, Olanzapine (OLZ) has been shown to effectively treat both positive and negative symptoms with minimal extrapyramidal symptoms, in addition to its association with improved antioxidant status [3,4]. However, OLZ’s mechanism of action, which involves blocking dopamine and serotonin receptors [5], is known to result in several side effects, including reduced locomotor activity [6], weight gain [7], sedation [8], and dizziness, which may result in new-onset diabetes and diabetic ketoacidosis in adults [9]. A hallmark of OLZ is that it significantly reduces locomotor activity from a single administration [10,11]. The reduction in the locomotor activity remaines during the day after the administration and as long as OLZ administration continues.

In this perspective, most studies focus on weight gain and metabolic syndrome as side effects of antipsychotics, with less focus on the reduction in locomotor activity. The decrease in locomotor activity significantly affects the quality of life of patients and can be considered a predisposing factor to other side effects related to OLZ treatment, such as metabolic syndrome [12,13]. This may exacerbate the patient’s mental disorder if left untreated [14]. Therefore, pharmaceutical therapies have been investigated to mitigate the negative effects of olanzapine (OLZ) medication. Betahistine, a histamine H3 receptor antagonist and H1 receptor agonist, has demonstrated efficacy in mitigating OLZ-induced weight gain and enhancing locomotor activity by alleviating some motor impairments linked to OLZ administration. Likewise, melanin-concentrating hormone (MCH) antagonists have shown the capacity to mitigate hypolocomotor consequences, considering MCH’s involvement in energy balance and locomotion regulation. Dopamine agonists have been evaluated for their potential to counteract OLZ-induced drowsiness and motor deficits; however, careful monitoring is essential to prevent disruption of OLZ’s antipsychotic efficacy. In addition, various non-pharmacological strategies have been suggested to alleviate the adverse effects linked to OLZ therapy. Lifestyle interventions, such as regular exercise and maintaining physical activity levels coupled with controlling food intake, can help mitigate the side effects of OLZ, complementing pharmacological treatments [6,15,16]. However, the negative effects of antipsychotics, in combination with the symptoms of mental illness, frequently discourage patients from participating in physical activity, leading to a reduction in their quality of life [6]. This study advances existing literature by introducing hyperbaric oxygen therapy (HBOT) as an innovative, non-pharmacological method aimed at addressing OLZ-induced hypomotility, hence providing a new therapeutic option for alleviating these motor deficits.

Thus, the overall aim of the current study is to address the locomotor impairment resulting from OLZ treatment in psychiatric patients in a rat model and introduce the utilization of hyperbaric oxygen therapy (HBOT) to alleviate OLZ-induced locomotor impairment in rats. HBOT is a therapeutic technique that is widely used in various medical fields [17]. It involves breathing pure oxygen in a pressurized chamber, which allows for increased oxygen dissolution into the blood plasma, resulting in positive biochemical, cellular, and physiological effects [18]. HBOT has been utilized in a range of clinical disorders, especially those related to hypoxia [19]. Furthermore, it has been applied to promote wound healing, injury recovery, and improve cognitive and locomotor performance, due to its proven positive effect on oxidative stress [20,21].

## 2. Materials and Methods

### 2.1. Animals and Experimental Design

Adult female and male Sprague Dawley rats were housed individually in a light–dark cycle, with lights on at 07:00 a.m., at a controlled temperature of 19–22 °C and humidity of 30–40%. The rats had ad libitum access to water and a standard rat diet. The animal experimental protocols were approved by the Animal Care Use Committee at Jordan University of Science and Technology which follows the animal care and uses guidelines (ILAR) (Institute of Laboratory Animal Resources U.S (1996). Guide for the care and use of laboratory animals. Washington, DC, USA: National Academy Press) (Institute of Laboratory Animal Resources (U.S.), (1996)) [22].

### 2.2. Drugs

Olanzapine powder with a purity of 99.2% (gift from Al-Hikma Pharmaceuticals, Amman, Jordan) was dissolved in 0.1 N HCL in distilled water. The resulting solution was adjusted to a pH of 5.5 with 0.01 N NaOH and then diluted with PBS to reach the required concentration of 1 mg/mL. The rats were injected intraperitoneally with olanzapine (or its vehicle) at a dose of 10 mg/kg for the time-course experiment, or for 14 consecutive days for the chronic experiment as described previously [23].

### 2.3. Oxygen Therapy (HBOT) Conditions

The oxygen therapy (HBOT) was administered to the rats daily for 14 days, for a duration of 2 h each day. The chamber’s oxygen concentration was maintained at 90–95%, and the pressure was gradually adjusted to reach 0.3 MPa (3 ATA) over a period of 20 min. The rats were treated with HBOT before OLZ injection every day, during the period between 8:00 a.m. and 11:00 a.m. The therapy duration was determined after the HBOT chamber was purged with pure oxygen from an oxygen generator and the chamber reached the desired pressure.

### 2.4. Habituation and Training on Rotarod Experiments

To prepare the animals for the experiment, they were habituated and trained on a rotarod (Woodland Hills, CA, USA). All experiments were conducted in a semi-sound-proofed room to attenuate the possible effects of uncontrolled variables such as loud sounds. On the first day, the animals were introduced to the experiment room. Then, animals received two days of training on the rotarod to become familiar with it before the start of the experiment. During the experiment, the animals were placed on a rod that initially moved at a speed of 4 rpm and gradually increased to a maximum of 40 rpm over a period of 300 s at fixed-rate acceleration, as described previously [24]. Multiple paper towels, that were changed between subjects, were placed beneath the rotating rod to absorb any secretions from animals and to provide a cushion in case the rat fell. The animals’ ability to stay on the device was recorded, reflecting their locomotor activity. The dependent variable measure was the amount of time the animal was able to stay on the rotarod until it fell off. These measures were recorded by an observer when the animal fell, or when 300 s was over. Baseline readings for each animal were taken prior to the administration of treatments.

Standardization, or time-course, experiments were conducted to determine the effective dose of OLZ that affects locomotor activity. In the time-course experiment, 5 female and 6 male rats were injected with a single dose of OLZ, and their locomotor activity was monitored using the rotarod until it returned to baseline readings. Thus, rotarod performance was recorded at 30 min, 60 min, 120 min, 5 h, 24 h, 48 h, and 72 h after OLZ injection or its vehicle. Rats were randomized to receive OLZ or vehicle in a crossover paradigm to minimize individual variations and bias related to rotarod learning. The rotarod’s rat activity was assessed through a single trial per rat for each tested time point.

### 2.5. HBOT and Locomotor Activity in Rats Receiving 14-Day OLZ Injection

A total of 76 adult female rats were randomly divided into four main groups (as shown in Table 1): the control group (CR) received only the vehicle injection without exposure to HBOT or OLZ injection; the second group (HBOT) was exposed to HBOT and received the vehicle OLZ injection; the third group (OLZ) received only the OLZ injection; and the fourth group (HBOOLZ) received HBOT first and then the OLZ injection. Each group underwent daily locomotor activity assessment on the rotarod, with three trials conducted per rat at each tested time point. The average of these three trials was recorded for each individual rat. The data collected over the course of 14 days was organized into four intervals: 1–2 days, 3–6 days, 7–10 days, and 11–14 days.

### 2.6. Measurement of Total Antioxidant Capacity

At the end of each experiment, the rats were decapitated, and their heads were placed on a paper towel. Using a scalpel, a sagittal cut was made from the posterior inferior end of the skull to the frontal part. The halves of the skull were then separated using forceps, placed in Eppendorf tubes, and kept in a deep freeze (−80 °C) until analysis. The total antioxidant capacity was determined using a kit from Cloud-Clone Corp, Houston, TX, USA according to the manufacturer’s instructions. The absorbance of both the standard and the samples was read at 414 nm using a plate reader. The TAC of the sample extract was calculated by comparing its absorbance to the values derived from the standard curve.

### 2.7. Statistical Analysis

All values are displayed as mean ± S.E.M. The results were compared using a two-way analysis of variance (ANOVA) using https://www.statskingdom.com, accessed on 20 September 2024, and significant differences among means were tested using a Student’s *t*-test OpenEpi https://www.openepi.com/Menu/OE_Menu.htm, accessed on 20 September 2024. Only *p*-values less than 0.05 were considered statistically significant.

## 3. Results

### 3.1. The Olanzapine Effect on Locomotor Activity: Time Course

**Figure 1:**
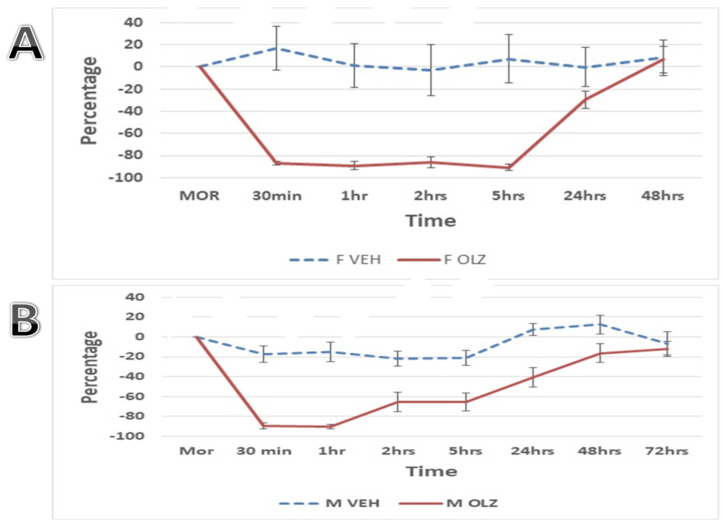
The impact of a single intraperitoneal injection of females (F OLZ; n = 5; Panel (**A**)) and males (M OLZ; n = 6; Panel (**B**)) with OLZ (10 mg/kg) or drug vehicle on rats’ activity on the rotarod. The rats’ locomotor activity was monitored until it returned to baseline levels for the time points presented on the x-axis. The percentage reflects the rotarod performance by averaging the readings from each time point and comparing to the baseline activity recorded for each animal at the end of its device adaptation period, before the administration of OLZ. The reading at each time point was obtained from one running trial on the rotarod device for each rat. Asterisks represent a significant difference compared to vehicle with a *p*-value less than 0.05, and the data are expressed as Mean ± SEM of percentages.

Subjects that were injected with the vehicle of OLZ did not exhibit any significant changes in their locomotor activity compared to pre-injection values at any time point in both male and female rats. However, the rats that received 10 mg/kg OLZ injection showed a marked decrease in their locomotor activity, experiencing a more than 88% reduction in both sexes. This reduction in the female rats persisted for 5 h before the animals’ locomotor activity began to partially recover at 24 h and completely recover after 48 h from OLZ administration (Figure 1A).

On the other hand, male rats exhibited a faster recovery of activity levels compared to females, such that they started to recover after 2 h after drug administration and stayed significantly different from vehicle-treated animals for 48 h. Male rats resumed their normal activity on the rotarod after 72 h from OLZ administration (Figure 1B). As mentioned earlier, the time-course experiment in male rats was conducted in a crossover study design to account for physical differences, but no significant variations were found among both subgroups of animals, as they all responded similarly to OLZ injection.

### 3.2. Effect of HBOT on Locomotor Activity in Female Rats Receiving Daily OLZ Injection

The two-way ANOVA performed on the four experimental treatment groups (CR, HBOT, OLZ, and HBOOLZ), with measurements taken daily at 30 min, 60 min, 120 min, and 24 h post-treatment, repeated over 14 days in intervals of 1–2, 3–6, 7–10, and 11–14 days, revealed a significant main effect of treatment on locomotor activity (F_(3, 1152)_ = 456.2, *p* < 0.00001), indicating substantial differences across groups. A significant effect of time intervals was also found (F_(15, 1152)_ = 9.27, *p* < 0.00001), suggesting that locomotor activity varied significantly across daily tested time points. Additionally, there was a significant interaction between treatment groups and time intervals (F_(45, 1152)_ = 1.93, *p* < 0.001), meaning the treatment effects on locomotor activity were dependent on the specific time intervals. These results demonstrate the dynamic interaction between treatment type and time on motor function, underscoring the potential of HBOT to modulate olanzapine-induced motor deficits over time.

**Figure 2:**
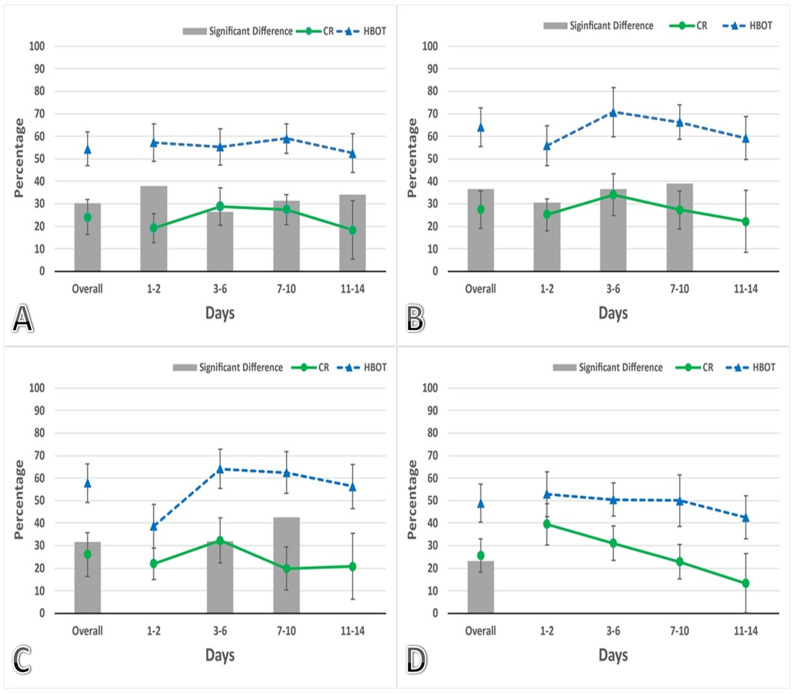
Effects of HBOT on female rats (HBOT group n = 14; CR group n = 14). Animals received vehicle injection for 14 days (divided into time intervals, (1–2), (3–6), (7–10), and (11–14), with daily intraperitoneal vehicle injections. The percentage reflects the rotarod performance by averaging the readings from the days indicated on the x-axis and comparing them to the baseline activity recorded for each animal at the end of the adaptation period, before the administration of HBOT. The reading at each time point was obtained as an average of 3 successive running trials for each rat on the rotarod device. The time points tested after OLZ injection were (**A**) 30 min, (**B**) 60 min, (**C**) 120 min, and (**D**) 24 h. Data are expressed as a Mean ± SEM of percentages. Gray bars are displayed only when the differences between groups reach statistical significance, with a *p*-value below 0.05.

Consequently, a key post hoc analysis focused on the effects of HBOT on locomotor activity, particularly its impact independent of olanzapine (OLZ) treatment (Figure 2A–D). In the control (CR) group, animals were not exposed to HBOT, but they were injected with a vehicle. These rats demonstrated an adaptation to the rotarod device, showing enhanced activity across the 14-day trial at multiple times (30 min, 60 min, 120 min, and 24 h) (*p* < 0.05). The animals’ rotarod performance in both groups showed a notable improvement during the initial time interval compared to the baseline recorded on the final day of the adaptation period. This enhanced performance remained stable and comparable throughout the remaining testing days. Overall, regardless of the day intervals tested, the animals demonstrated a 24% to 27% improvement in rotarod performance relative to their initial recorded time. On the other hand, the group receiving HBOT demonstrated significantly higher activity levels compared to the control group at several time points on different time intervals (*p* < 0.05). Their overall activity levels ranged from 48% to 64% higher than the time recorded on the final day of the adaptation period, representing nearly double the improvement seen in the control group.

**Figure 3:**
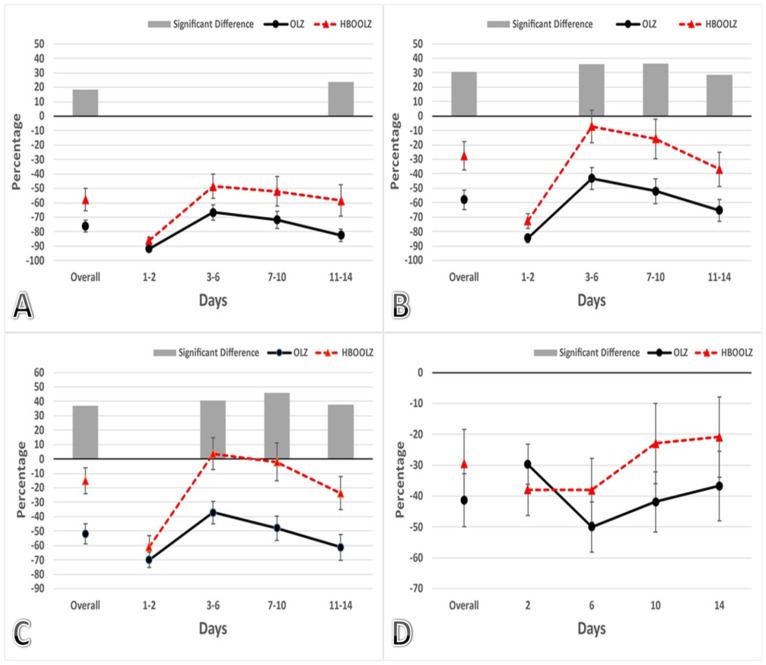
Effects of HBOT on rotarod activity on animals that received 10 mg/kg OLZ for 14 days (divided into blocks of (1–2), (3–6), (7–10), and (11–14). The percentage reflects the rotarod performance by averaging the readings from the days indicated on the x-axis and comparing them to the baseline activity recorded for each animal at the end of the adaptation period, before the administration of OLZ or HBOT. The reading at each time point was obtained as an average of 3 successive running trials for each rat on the rotarod device. The time points tested after OLZ injection were (**A**) 30 min, (**B**) 60 min, (**C**) 120 min, and **(D**) 24 h. Data are expressed as a Mean ± SEM of percentages. Gray bars are displayed only when the differences between groups reach statistical significance, with a *p*-value below 0.05.

The core of the study aimed to assess the impact of HBOT on locomotor activity in animals receiving OLZ. Another post hoc comparison was made between the OLZ group and the HBOOLZ group over a period of 14 days (Figure 3). The results showed an overall improvement in locomotor activity during the initial time points tested (30, 60, and 120 min) following daily OLZ injections (*p* < 0.05). Interestingly, HBOT did not lead to any improvements in locomotor activity for OLZ-treated animals during the first two days (1–2 days) at any of the tested time points (Figure 3). However, the positive effects of HBOT on locomotor activity in OLZ-treated animals showed time-dependent improvements on day 3 onward (*p* < 0.05). At the 30 min time point, significant improvements were noted only after prolonged treatment (days 11–14) (Figure 3A) (*p* < 0.05), while at the 60 and 120 min time points after OLZ treatment, effects were noticeable starting from day 3 onwards (Figure 3B,C) (*p* < 0.05). In some instances, the locomotor activity even returned to normal levels with the assistance of HBOT (3–6 and 7–10 time intervals). It is important to note that at all tested time points and across all day intervals, the CR and HBOT groups consistently showed significantly higher activity levels compared to the rats receiving OLZ, regardless of HBOT’s combination with OLZ (*p* < *0.05*). This is represented when statistically comparing the groups shown in Figure 2 and Figure 3.

### 3.3. The Effect of OLZ and HBOT on the Total Antioxidant Capacity

**Figure 4:**
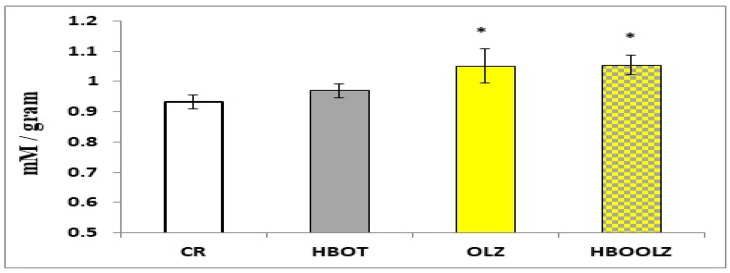
TAC as a result of 14 days of treatment assessed in brain homogenized samples. Data are expressed as a Mean ± SEM of percentages. The significant difference with a *p*-value less than 0.05 is represented by asterisks in comparison to control (CR).

The brain tissue homogenates from all experimental groups were analyzed to measure the total antioxidant capacity (TAC). The results revealed a significant increase in TAC in both the HBOOLZ and OLZ groups (*p* < 0.05). However, no significant difference in TAC was observed between the HBOT group and the CR group (Figure 4).

## 4. Discussion

There have been no prior investigations examining the potential benefits of hyperbaric oxygen therapy (HBOT) in alleviating the adverse effects on locomotor activity caused by OLZ treatment. Thus, this study showed three main findings. First, acute injection of OLZ produced significant locomotor reduction that may last up to 48–72 h in female and male rats, respectively. Second, partial tolerance to this effect may develop after 3 days from chronic OLZ administration. HBOT co-treatment with OLZ helped in attenuating OLZ-induced hypolocomotion. Finally, results of this study indicated that HBOT did not affect the therapeutic efficacy of OLZ in animal subjects on total antioxidant capacity (TAC) levels, as OLZ treatment typically leads to an increase in TAC levels, contributing to the reduction in oxidative stress, which is considered one of the potential pathophysiological causes of schizophrenia [3]. All together, these results suggest the beneficial use of hyperbaric oxygen with OLZ treatment to decrease its motor impairment effect, thus increasing patients’ adherence by decreasing associated side effects.

In the animal model, OLZ decreased the animals’ locomotor activity with a reduction reaching 40% [25], which is consistent with our findings in this study. This decrease continued throughout the day and as long as the drug was taken. Moreover, we showed that female rats showed a robust decrease in locomotor activity for 5 h, and they needed 48 h to return to their normal locomotor activity (Figure 1A). However, male rats showed gradual improvement across the 72 h with the worst activity being reported for only one hour after drug injection (Figure 1B). The observed difference in hypolocomotor activity between female and male rats may align with previous clinical findings, where antipsychotic side effects such as weight gain, metabolic syndrome, tardive dyskinesia, agranulocytosis, and cardiac arrythmia tend to manifest more prominently in females that were treated with antipsychotic drugs compared to male subjects [26,27,28]. In the current study, female rats exhibited greater hypolocomotor activity with OLZ treatment, leading us to prioritize investigating the potential of hyperbaric oxygen therapy (HBOT) to mitigate these side effects. This particular side effect in different sexes is usually overlooked in animal models treated with OLZ, thus making this current work the first study to clearly compare the dynamic of this side effect in both sexes. Despite this, most preclinical studies have traditionally been conducted on male rats, as they allow for simpler experimental designs and avoid the variability introduced by the estrous cycle in females, which can affect drug pharmacokinetics and pharmacodynamics [29]. Therefore, the current work clearly demonstrated using male animals to test antipsychotic drug performance may be inaccurate and inappropriate for females as differences in response, at least for the side effect on locomotor activity, do exist. It is important to mention that this decrease in locomotor activity may be connected and predisposed to weight gain, as reported by previous human studies [26,30].

In the time-course experiment, only one trial of rotarod was conducted at each time point (30 min, 1 h, 2 h, 5 h, and 24 h) which added up to be five trials on the rotarod per animal per 24 h, while in the subsequent 14-day experiments, three trials were performed for each rat then averaged to represent the reading at each time point. Therefore, each animal in all experimental groups underwent three running rounds (trials) that were replicated four times within a 24 h period (at 30, 60, 120 min, and 24 h) for 14 days, which makes the total trails per 24 h, 12 running trials. As a result, control subjects in the 14-day experiment showed improved locomotor activity in the rotarod experiment compared to the time-course experiment (Figure 1), surpassing their initial recorded times by on average of around 30% (Figure 2). This observation helps to explain the perceived enhancements in animals receiving only OLZ treatment for 14 days. Furthermore, it sheds light on the reason why animals that ran more rounds on the rotarod were able to recover their normal locomotor activity within 24 h (Figure 3D), rather than 48 h as was documented in the time-course experiment (Figure 1). These findings suggest that increasing the number of running rounds on the rotarod device positively contributes to mitigating the side effects of locomotor impairment. Additionally, during the standardization experiments, as shown in Figure 1, we observed that the animals returned to their baseline locomotor activity within the first 48 h. This observation justified combining the data for the initial time intervals (1–2 days) in the subsequent experiments presented in Figure 2 and Figure 3. For the remaining intervals shown in these figures, the data were grouped into four-day intervals rather than two-day intervals to reduce clutter, as no significant differences were observed between the combined intervals.

Antipsychotic medications are known to block dopamine receptors, resulting in noticeable motor impairment that significantly affects motor coordination and the initiation of movement [10]. The rotarod test provides measurements such as the time spent in movement, which can be reflected on the distance traveled on a rotating cylinder, making it a valuable tool for assessing locomotor activity [31]. Also, it can also be considered a form of forced exercise that also might affect anxiety and alter spatial learning/memory [32]. Therefore, the improvement of locomotor activity that was noticed during the current experiment, specifically in the CR group, indirectly emphasizes the effect of exercise alone in improving the locomotor activity, which might be an additional factor in the current experiment that resulted in an alleviation of the hypolocomotor side effects that are associated with OLZ treatment. Consequently, it would be beneficial to compare the rotarod test with other established methods used for assessing locomotor activity in the existing literature, such as the open field test, which evaluates exploratory behavior and general activity in addition to motor activity [33]. Utilizing multiple assessment methods in future studies will provide a more comprehensive understanding of locomotor activity and its associated behaviors in the presence of HBOT.

To counteract the negative impact of OLZ on locomotor activity, patients are often advised to engage in physical activity due to its positive effects on improving their quality of life [34,35,36]. Patients who adhered to exercise have shown an improvement in their motor activity, which is also reflected in the OLZ-induced obesity [6]. This observation was also reported previously using an animal model, where exercise ameliorated the side effects by avoiding weight gain and glucose tolerance [37]. Also, exhaustive exercise but not moderate was able to protect against OLZ-induced hyperglycemia [38]. However, exercise may be an unfavorable choice for patients receiving antipsychotics, which explains the lack of comprehensive studies about this intervention in the literature [6]. For instance, in schizophrenia patients, engaging in physical activity may seem unattainable due to a lack of motivation, stress, mood instability, and lack of social support [39,40]. In addition, these challenges urge patients and physicians to develop new strategies to improve the quality of life of patients taking OLZ chronically.

HBOT is a safe therapy for several troublesome diseases that require improving physical activity, mental and physical health, and enhancing social performance [41]. It has anti-oxidative, anti-inflammatory, neuroprotective, and anti-apoptotic properties [42]. The present study utilizes HBOT as an alternative, more manageable therapy to mitigate the reduction in locomotor activity caused by OLZ treatment. The findings indicate that HBOT can alleviate OLZ-induced locomotor side effects as early as 30 min post-administration, with significant improvements observed after 11 days of continuous OLZ treatment (Figure 3A). Notably, the beneficial effect of combining HBOT and OLZ (HBOOLZ group) became evident after 3 days of HBOT application, particularly at 60 and 120 min following daily OLZ administration (Figure 3B,C).

Oxidative stress is known to play a significant role in the pathophysiology of schizophrenia, cognitive decline, and behavioral abnormalities [43]. While it is not the sole cause of these conditions, research has indicated that individuals with schizophrenia often exhibit reduced levels of antioxidant enzymes [44]. Interventions and medications targeting oxidative stress have been suggested as potential approaches for schizophrenia treatment [45]. Studies have observed increased antioxidant enzyme activity in serum samples collected from OLZ-treated patients, suggesting OLZ’s potential in mitigating the negative impact of oxidative stress commonly associated with schizophrenia [3,46]. However, some previous reports indicated that long-term OLZ use may induce oxidative stress, necessitating the monitoring of oxidative stress levels [47,48]. Overall, managing OLZ-induced oxidative stress remains complex, as short-term OLZ use may boost antioxidant defenses, but extended use has been linked to mitochondrial dysfunction and increased production of reactive oxygen species (ROS), which can overwhelm the body’s natural defense [47,48,49]. It is intriguing to note the interplay between HBOT and exercise in relation to oxidative stress. Human and animal studies have demonstrated that regular exercise can initially induce oxidative stress but ultimately adapt to this stress by upregulating antioxidant enzymes [50]. This adaptation is reflected in reduced oxidative stress markers and increased antioxidant markers [51].

After 14 days of daily administration of OLZ to rats at a high dose in the current experiment, the TAC seems to be upregulated in this particular group compared to the CR group (Figure 4). This highlights the importance of monitoring oxidative stress levels during OLZ treatment. On the other hand, HBOT offers a non-pharmacological approach that also has conflicting results in regard to oxidative stress and antioxidant capacity. In several studies, it was reported that HBOT does not directly affect systemic TAC but promotes tissue repair and oxygenation, helping to mitigate oxidative damage [52] and has been shown to reduce ROS and inflammation in tissue healing [52]. During our HBOT experiments, our aim was not to nullify the effectiveness of OLZ by delaying its initial antioxidant properties. We even expected HBOT to improve antioxidant status; however, this outcome was not achieved. Our data revealed a significant increase in total antioxidant capacity after OLZ treatment that was not affected when HBOT was co-administered in the HBOOLZ group (Figure 4). A likely explanation is the use of the rotarod to measure locomotor activity, which essentially acts as a form of exercise for the animals. Regular physical exercise, despite initially raising oxidative stress, leads to a long-term adaptive response by upregulating antioxidant enzymes, further reducing oxidative damage [53]. Since exercise is well-documented to upregulate antioxidant production, it may have elevated the baseline TAC levels, masking any additional effects of HBOT. Nevertheless, we were able to demonstrate that the combination of HBOT and exercise, achieved by running on rotarod in the current experiment, synergistically enhanced the beneficial properties of OLZ, which probably improved the side effects associated with OLZ without compromising its pharmacological efficacy.

As stated earlier, the role of oxidative stress in the pathogenesis of schizophrenia has been recognized, as elevated oxidative stress markers and reactive oxygen species (ROS) were prominent in patients [54]. This is thought to contribute to neurodegeneration and cognitive decline in schizophrenia, complicating the disease’s progression. Enhancing antioxidant capacity could play a fundamental role in mitigating this damage, as increased antioxidant levels can specifically help neutralize ROS and protect neural tissues [55]. In this context, HBOT may not only counter hypoxia but may also offer potential benefits by reducing oxidative stress and promoting cellular repair [52]. This makes it a valuable complementary treatment option to olanzapine, for which the antioxidant activity seems to eventually fade with time and more likely exaggerate the oxidative stress resulting from prolonged OLZ treatment [47,49].

Finally, assessing the feasibility of implementing HBOT in clinical settings is vital, with careful consideration of its socioeconomic impact and patient acceptance. As this is the first study proposing HBOT as part of the treatment protocol for schizophrenic patients to subside side effects, further clinical trials are essential to establish a formalized HBOT protocol, particularly in conjunction with OLZ treatment. These trials may indicate the importance of HBOT during the early stages of OLZ therapy, when patients are more vulnerable to side effects, and suggest that, as patients adapt to the medication (OLZ), the frequency of HBOT sessions could be gradually reduced to once or twice per week. Additionally, the option of personalized HBOT chambers for home use, allowing for short daily sessions (10–15 min), could offer a more accessible and less physically demanding alternative to the intense exercise usually suggested to schizophrenia patients, which may not be practical or appealing for them.

In summary, the utilization of HBOT has the potential to decrease the adverse effects on locomotor activity associated with OLZ and enhance the overall well-being of patients. It is interesting to note from the existing literature that there are numerous shared beneficial properties between HBOT and exercise which are usually recommended to schizophrenic patients treated with OLZ. These advantages could be implemented not only as a supplement to antipsychotic treatment but also to enhance the patient’s quality of life and potentially address the underlying mechanisms of mental illness. We recommend conducting further research to establish the safety and efficacy of HBOT in human subjects, with the aim of alleviating the negative impact of OLZ on locomotor activity, which significantly influences patients’ quality of life and overall wellness.

## 5. Conclusions

This study provides compelling evidence supporting the therapeutic potential of HBOT in ameliorating the reduction in locomotor activity induced by OLZ administration. The findings underscore the importance of exploring adjunctive treatments such as HBOT to enhance the overall efficacy and tolerability of antipsychotics, ultimately improving outcomes for patients with mental illnesses.

## Figures and Tables

**Table 1:** Experimental design to assess the effect of HBOT on locomotor activity in SD rats receiving OLZ injection.

Groups	Abbreviations	HBOT	OLZ Injection	Animals Number
Control	CR	None	None	14
HBOT only	HBOT	Present	None	14
OLZ	OLZ	None	Present	24
HBOT + OLZ	HBOOLZ	Present	Present	24

## Data Availability

The data presented in this study are available upon request from the corresponding author.
